# Sleep disorders and psychological comorbidities in women with polycystic ovary syndrome – a cross-sectional study

**DOI:** 10.1007/s00404-025-08049-9

**Published:** 2025-05-13

**Authors:** Claire Decrinis, Konstantin Hofmann, Norman Bitterlich, Adrian Singer, Katharina Tropschuh, Serena Lozza-Fiaco, Julia Estermann, Annette Bachmann, Petra Stute

**Affiliations:** 1 Faculty of Medicine, Department of Obstetrics and Gynecology, University of Freiburg Medical Center, Hugstetter Straße 55, 79106 Freiburg, Germany; 2 Department of Obstetrics and Gynecology, Mainz University Medical Center, Mainz, Germany; 3 Statistics – Analysis, Consulting, Training DE in Connection with www.statistik-abw.de, Chemnitz, Germany; 4 Department of Obstetrics and Gynecology, University Hospital Zurich, Zurich, Switzerland; 5 Division of Gynecology and Obstetrics, Klinikum Rechts Der Isar, Technical University of Munich, Munich, Germany; 6 Department for Gynecologic Psychiatry, Psychiatry St. Gallen, St. Gallen, Switzerland; 7 Department of Anesthesiology and Intensive Care, Baden Cantonal Hospital, Baden, Switzerland; 8 Division of Gynecological Endocrinology and Reproductive Medicine, Department of Gynecology and Obstetrics, University Hospital Frankfurt, Frankfurt, Germany; 9 Department of Obstetrics and Gynecology, University Clinic Inselspital Bern, Bern, Switzerland

**Keywords:** Polycystic ovary syndrome, Affective disorder, Depression, Anxiety, Sleep disorder, Obstructive sleep apnea

## Abstract

**Purpose:**

Polycystic ovary syndrome (PCOS) is a metabolic and hormonal disorder that affects physical and emotional well-being. The aim of this cross-sectional study was to assess associated factors like sleep disturbance, obstructive sleep apnea (OSA), anxiety and depression in a German-speaking population with PCOS.

**Methods:**

We designed an anonymous online survey with items from validated questionnaires, including the Hospital Anxiety and Depression Scale (HADS), the Generalized Anxiety Disorder (GAD-7), the Pittsburgh Sleep Quality Index (PSQI) and the STOP-Bang questionnaire to screen for OSA. The survey was mainly distributed via social media in Austria, Germany and Switzerland. Data from 587 questionnaires were analyzed.

**Results:**

Based on the STOP Bang questionnaire, 19.5% of women had a high probability for OSA. BMI and insulin resistance were identified as independent associated factors with OSA (both p < 0.001). Overall, the median anxiety score (GAD-7) was in the moderate range (Median 10.0, Interquartile range (IQR) 8.0). According to the HADS, association with moderate to severe anxiety (HADS-A) was 52.0% and with moderate to severe depression (HADS-D) 27.8%. There was a significant positive correlation between HADS-A/ HADS-D and BMI (r = 0.122, (HADS-A)/ r = 0.223 (HADS-D), both p < 0.01). According to the PSQI, 60.5% had mild sleep disturbance and 29.7% had chronic sleep disturbance. Chronic sleep disturbance was associated with anxiety disorders and depression, as well as a high probability of OSA (p < 0.001) after adjustment for age.

**Conclusion:**

Our study highlights the probability of depression, anxiety and sleep disorders, including OSA, in women with PCOS and their association with BMI and insulin resistance.

**Supplementary Information:**

The online version contains supplementary material available at 10.1007/s00404-025-08049-9.

## What does this study add to the clinical work

**Table:** 

This study underscores the high prevalence of anxiety, depression, and sleep disturbances in women with PCOS, especially for overweight patients and women with insulin resistance. There is a need for routine mental health and sleep disorder screenings in clinical practice.	

## Introduction

Polycystic ovary syndrome (PCOS) is a common endocrine disorder with a prevalence of 5–13% in the female population [1]. The number of unrecorded cases is estimated to be even higher. The main features of PCOS are hyperandrogenism, oligomenorrhoea and polycystic ovarian morphology [2]. In recent decades, it has been well established that PCOS does not only affect the reproductive system, but is a systemic, chronic disease associated with metabolic complications such as insulin resistance, diabetes [3], obesity and cardiovascular disease [4]. It is also associated with an increased risk of endometrial cancer [5], sleep disorders [6] and psychological comorbidities [7].

Although PCOS was first described in 1935 [8], the link between sleep disturbance and PCOS is relatively recent. As shown by Fernandez et al. [6] there is a small database on the subject with most papers having been published in the last 20 years, making it an interesting topic for further research. The most commonly reported sleep disturbance in women with PCOS is obstructive sleep apnea (OSA). OSA is defined as a collapse of the upper airway during sleep, resulting in periods of hypopnea and apnea phases with arousal from sleep and oxyhemoglobin desaturation [9]. PCOS is also associated with various sleep disturbances such as reduced sleep duration, poor sleep quality, fragmented sleep, difficulty falling asleep, and daytime sleepiness [10, 11]. These sleep problems can worsen other PCOS symptoms and contribute to a cycle of declining health. Therefore, addressing sleep problems is crucial to the overall management of PCOS.

Numerous studies [12, 13] and meta-analyses [7, 14] have shown that women with PCOS are at higher risk of depression and anxiety, with more severe symptoms compared to the general population in different countries. The recently published, updated European Society of Human Reproduction and Embryology (ESHRE) guideline [15] highlights the importance of screening all adolescents and adults with PCOS for these psychological comorbidities. Data from German-speaking countries are limited, with only one published study available [16].

The aim of this cross-sectional study was to determine the association of sleep disorders, anxiety and depression in German-speaking women with diagnosed or suspected PCOS. We hypothesized that these prevalences would be high and that there would be an association between sleep disturbances and metabolic complications and psychological comorbidities. We also wanted to evaluate possible weight-dependent associations between sleep disorders and depression or anxiety.

## Methods

We designed the cross-sectional study as an online survey to reach as many participants as possible. All answers and criteria were self-reported. Inclusion criteria were defined as having given informed consent to participate, being female, aged 18 years or older, and meeting the ESHRE criteria for PCOS [15]. We also included postmenopausal women in the study if they met the above criteria. Exclusion criteria were other causes of hyperandrogenism such as adrenogenital syndrome or prolactinoma/hyperprolactinemia, pregnant or breastfeeding participants, women without menarche, and women with thelarche less than three years previously. Being diagnosed with PCOS by a gynecologist but not meeting the ESHRE criteria was also a reason for exclusion. As the questionnaire was in German and online, sufficient language skills and internet access were mandatory. If any of the exclusion criteria were met, the questionnaire was terminated early, and the participant was informed of the exclusion.

### PCOS inclusion criteria

The participants had to meet the ESHRE criteria for PCOS, which require at least two of the following three features after excluding other etiologies: oligo/anovulation, clinical and/ or biochemical hyperandrogenism, and polycystic ovarian morphology on ultrasound [15]. All criteria were asked in the questionnaire; however, the diagnosis in our study was based on self-reported answers. The authors did not have access to medical records or laboratory and ultrasound exams.

### Questionnaire

The questionnaire was programmed using SoSciSurvey, a platform whose server is located in Germany. Therefore, data collection fell under the General Data Protection Regulation (DSGVO) and high security standards of data encryption were ensured. All data were collected anonymously. To minimize double counting, an individual identification code was created at the beginning of the survey, consisting of defined letters of the parents’ name, the own name and the year of birth.

Subjects were recruited through online forums, social media, newsletters, self-help groups, websites and consultations in hospitals and doctors’ offices in Austria, Germany and Switzerland. The recruitment period was from 15th of November 2023 to 5th of February 2024. The study was approved by the Ethics Committee of the Canton of Berne (BASEC Req-2023-01259) and all methods were performed in accordance with the relevant guidelines and regulations. According to the study protocol, a formal ethical approval was not required due to the anonymous data collection. The letter of approval is available as a supplementary file (File S1).

The questionnaire was adapted from previous publications [17, 18]. The German version of validated questionnaires was included in our self-constructed questionnaire. Relevant validated questionnaires for assessing at-risk prevalence of depression and anxiety, sleep quality and obstructive sleep apnea were the Hospital Anxiety and Depression Scale (HADS) [19], the Pittsburgh Sleep Quality Index (PSQI) [20], the STOP-bang questionnaire [21] and the Generalized Anxiety Disorder (GAD-7) questionnaire [22].

Other areas covered by the questionnaire were PCOS diagnostic criteria, demographics, aesthetic aspects such as hirsutism, acne and alopecia, menstrual cycle profile, metabolic comorbidities, and reproductive aspects. The full questionnaire and codebook are included as supplementary files (Files S2, S3).

## Specific Items

### Pittsburgh sleep quality index (PSQI)

The PSQI has been utilized by several studies to evaluate sleep disturbances in women with PCOS, demonstrating its reliability and applicability in this population [23]. It consists of 19 self-assessment questions and five questions to be answered by a partner or roommate. Only the self-assessment questions are included in the total score and are grouped into seven sub-domains. Each sub-domain can have a value from zero (no problems) to three (severe problems), resulting in a total score from zero to 21. Healthy sleepers typically score five points or less, while mildly disturbed sleep is characterized by a range of six to ten points. A score of 11 and above indicates chronic sleep disturbance [20].

### Hospital anxiety and depression scale (HADS)

The HADS questionnaire is validated for patients with polycystic ovary syndrome. Multiple studies have utilized the HADS to assess anxiety and depression levels in women with PCOS, Proving its effectiveness and dependability within this patient population [24, 25]. ´It consists of 14 questions divided into two domains: the HADS-D (depression) and the HADS-A (anxiety). Each domain has a maximum score of 21 points, with a cut-off score of eight. A score between eight and ten indicates a chance of mild depression or anxiety, 11 to 14 equals to a chance of moderate depression or anxiety, and 15 and above indicates a chance of severe depression or anxiety [19].

### STOP-bang questionnaire

The STOP-bang questionnaire classifies the probability of OSA into low (zero to two questions answered with yes), moderate (three to four questions answered with yes) and high (five to eight questions answered with yes or at least two of the first four questions answered with yes plus being male or having a BMI > 35 kg/m^2^ or a neck circumference greater than 43 cm for men or 41 cm for women) [21]. The STOP-BANG questionnaire has been validated in midlife women, showing good predictive ability for OSA, with a sensitivity of 77% and specificity of 45% for moderate to severe OSA [26]. Given the high prevalence of OSA in women with PCOS and the metabolic and endocrine disturbances that increase OSA risk, the STOP-BANG questionnaire is a practical and validated tool for this population.

### GAD-7

The GAD-7 is a questionnaire to assess generalized anxiety disorder (GAD) and is recommended by the ESHRE guidelines to screen for anxiety in PCOS, as it has been validated in this population as an accurate screening tool [15, 27]. Participants rate their answers from zero (not affected) to three (highly affected). The sum of all seven questions gives the GAD-7 score, with a score of zero to four indicating minimal anxiety symptoms and five to nine corresponding to mild anxiety symptoms. A score of ten to 14 indicates moderate anxiety, and a score of 15 or higher equals to severe anxiety [22].

## Statistics

To detect differences in binomial proportions and small effects in scores (with a Cohen’s effect size of at least 0.25) with 80% power, a sample size of at least 200 participants was calculated. All data collected were analyzed descriptively using frequency distributions (nominal and ordinal variables) or statistical characteristics such as median, quartiles and range (continuous or ordinal variables). Normal distribution was tested using the Shapiro wilk test. Since the presence of normal distributions could not be confirmed, all statistical tests were non-parametric. The questionnaires were analyzed according to published protocols. We distinguished between raw scores (scores that are the sum of the responses to the items, considered as continuous variables) and score values (three- to four-level categorization, considered as ordinal variables).

Correlations between continuous parameters were tested using the non-parametric Spearman-Rho test. Results were supplemented with a linear regression model, both univariate and multivariate, adjusted for age and sex, and the F value was reported for significant explanatory power. Differences between score categories were tested for proportions using the Chi^2^ test and for continuous variables using the Kruskal–Wallis test (more than two independent groups) or the Mann–Whitney U test (pairwise comparisons).

A significance level of p < 0.05 was considered statistically significant. Exploratory data analysis did not consider the effects of multiple testing. Missing data were indicated as such and were not replaced.

Data were analyzed using SPSS software version 26.0 and graphs were generated using GraphPad Prism version 10 or Microsoft Excel.

## Results

**Fig. 1:**
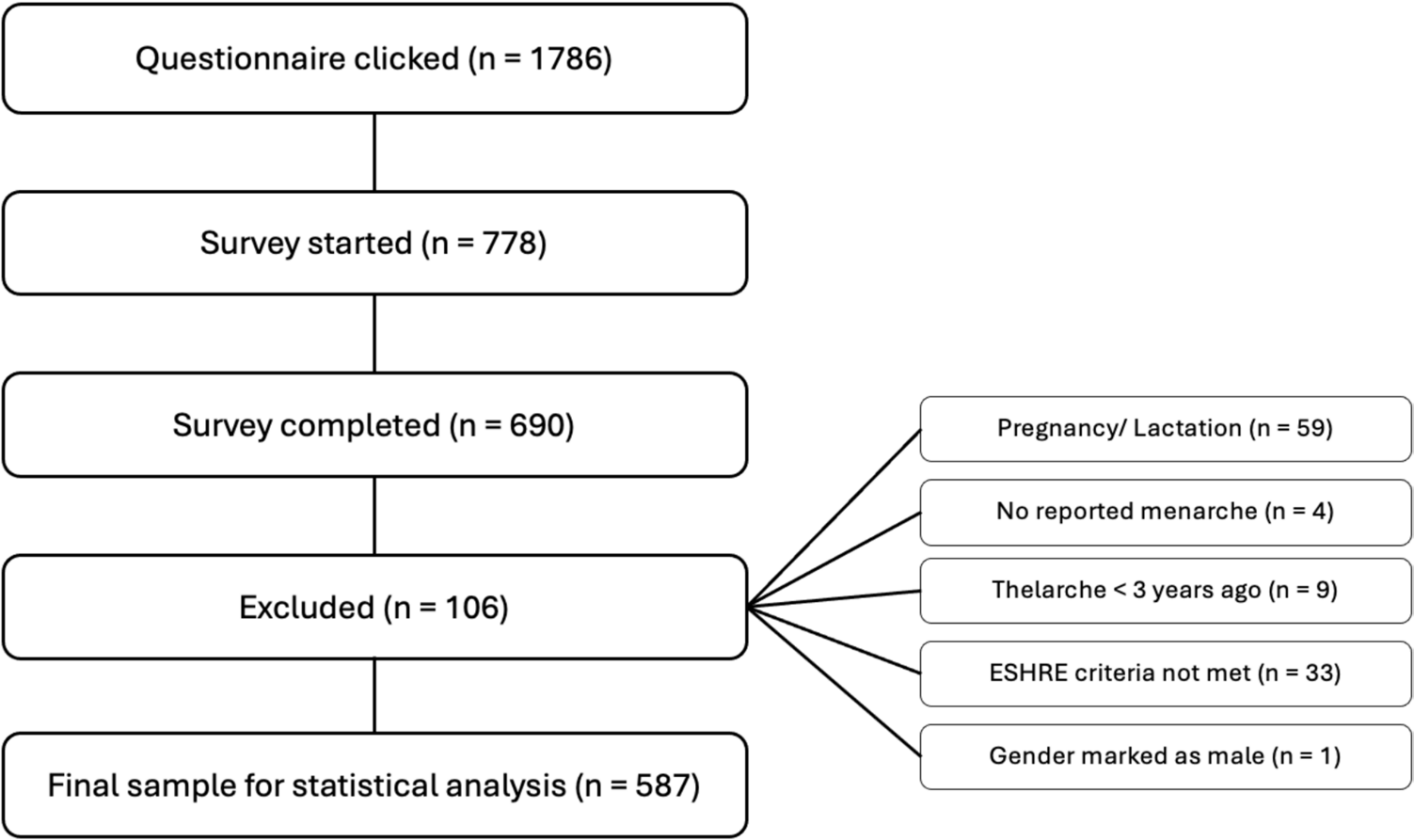
Participant eligibility and exclusion criteria

The questionnaire was clicked on 1786 times and 778 people started the survey, but only 690 completed it. After exclusion, 587 records were included in the statistical analysis (Fig. 1).

### Characteristics of the study population

Participants ranged in age from 20 to 60 years, with a median age of 32.0 years, and an interquartile range (IQR) of 7.0 years. A total of 323 participants (55.0%) were obese according to the WHO definition (BMI ≥ 30 kg/m^2^), whereas only 134 participants (22.9%) were of normal weight (BMI 18.5–25 kg/m^2^). Most of the women lived in Germany (91.3%) and were married or in a relationship (83.1%). Focusing on metabolic comorbidities, a small number of participants had manifest diabetes type I or II (2.7%), but almost half were at high risk of developing manifest diabetes (self-reported insulin resistance) (45.5%). A total of 230 participants (39.2%) reported taking medication for diabetes. 12.4% of participants had arterial hypertension (N = 73), other common comorbidities were hypothyroidism (11.2%, N = 66) and hypercholesterolemia or fatty liver disease (6.9%, N = 40). Aesthetic complaints due to hyperandrogenism were common: 53.7% (N = 315) of participating women reported acne, 45.7% (N = 268) reported alopecia according to the Ludwig classification [28] and 81.1% (N = 476) complained about hirsutism (score > seven on the Ferriman-Gallwey questionnaire [29]) (Table 1).

**Table 1:** Characteristics of the study population

Characteristic	Value*
Age of participants – median, IQR	32.0, 7.0
BMI – median, IQR (kg/m^2^)	31.4, 12.2
Underweight (BMI < 18.5)	5 (0.9%)
Normal weight (BMI 18.5 – 24.9)	134 (22.9%)
Overweight (BMI 25—29.9)	124 (21.2%)
Obese (BMI > 30)	323 (55.0%)
Country of origin	
Germany	536 (91.3%)
Austria	26 (4.4%)
Switzerland	16 (2.7%)
Others	9 (1.5%)
Marital status	
Single	92 (15.7%)
Married	263 (44.8%)
In a partnership	225 (38.3%)
Divorced	7 (1.2%)
Highest level of education	
Currently attending school	1 (0.2%)
School-leaving qualification	37 (6.3%)
Secondary school certificate	148 (25.2%)
Polytechnic secondary school	4 (0.7%)
Advance college certificate	85 (14.5%)
High school diploma	105 (17.9%)
University diploma	186 (31.7%)
Other	21 (3.6%)
Smoking	
Yes	139 (23.7%)
No	448 (76.3%)
Types of diabetes mellitus	
Manifest Diabetes mellitus	16 (2.7%)
Gestational diabetes	30 (5.1%)
Insulin resistance	265 (45.5%)
None	274 (46.7%)
Other comorbidities	
Arterial Hypertension	73 (12.4%)
Hypothyroidism	66 (11.2%)
Hypercholesterolemia/ Fatty liver disease	40 (6.9%)
Endometriosis	11 (1.9%)
Migraine	12 (2.0%)
Acne	
Yes	315 (53.7%)
No	247 (42.1%)
Don’t know	25 (4.3%)
Alopecia	
Yes	268 (45.7%)
No	319 (54.3%)
Hirsutism	
Yes	476 (81.1%)
No	82 (14.0%)
Don’t know	29 (4.9%)
Menopause status	
Premenopausal	569 (96.9%)
Postmenopausal	18 (3.1%)
Fertility-Problems (Have you ever tried to get pregnant for more than one year?)	
Yes	285 (48.6%)
No	302 (51.4%)

*No. of participants (%), if not otherwise specified. Abbreviations: *BMI* Body Mass Index, *ESHRE* European Society of Human Reproduction and Endocrinology, *IQR* interquartile range

### Anxiety was the predominant psychological comorbidity in women with PCOS

Anxiety was the most common psychological comorbidity in women with PCOS: 52.0% of participants (N = 305) scored 11 or more on the HADS-A, indicating a chance of moderate to severe anxiety. The median score for the entire study population was 11 points (IQR 6.0). When these results were compared with the GAD-7, similar results were found. 32.2% (N = 189) scored 10 to 14 on the GAD-7, equaling a chance of moderate anxiety, while 25.0% of participants (N = 147) had an association with severe anxiety (score 15 to 21 on the GAD-7).

27.8% of the women (N = 163) scored 11 or higher on the HADS-D, indicating a high chance of moderate to severe depression (median 8.0, IQR 6.0). BMI (in kg/m^2^) correlated significantly with the HADS Anxiety and HADS Depression scales (HADS-A r = 0.122, p = 0.003, HADS-D r = 0.223, p < 0.001), indicating that overweight and obese as well as underweight women with PCOS had a higher association with anxiety and depression. For the HADS anxiety subgroup, these correlations were confirmed by a linear regression model with HADS anxiety scores as the dependent variable and BMI as the independent variable (F = 8.892; p = 0.003). However, when the regression model was adjusted for age and PSQI total score (F = 51.153; p < 0.001), the influence of BMI was not statistically significant. For the HADS depression subgroup, the regression model showed statistical significance even after adjusting for age and PSQI total score (F = 48.991; p < 0.001).

**Fig. 2:**
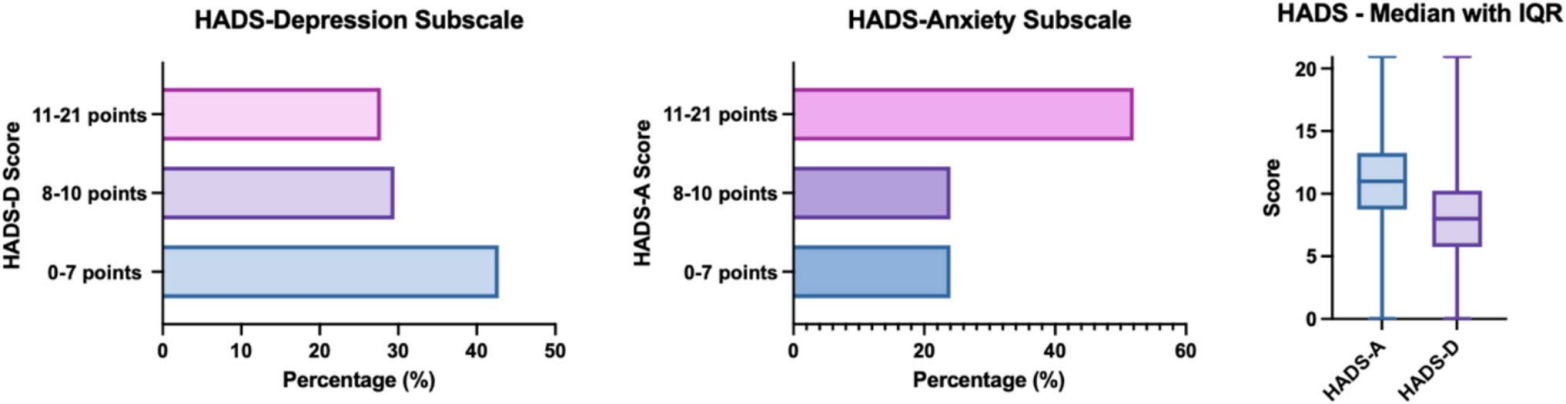
Frequency distribution of the Hospital Depression and Anxiety Score (HADS)

The HADS anxiety and depression scores (range zero to 21) correlated statistically significantly with the GAD score (range zero to 21) (HADS-A r = 0.772, p < 0.001, HADS-D r = 0.568, p < 0.001) (Fig. 2).

**Fig. 3:**
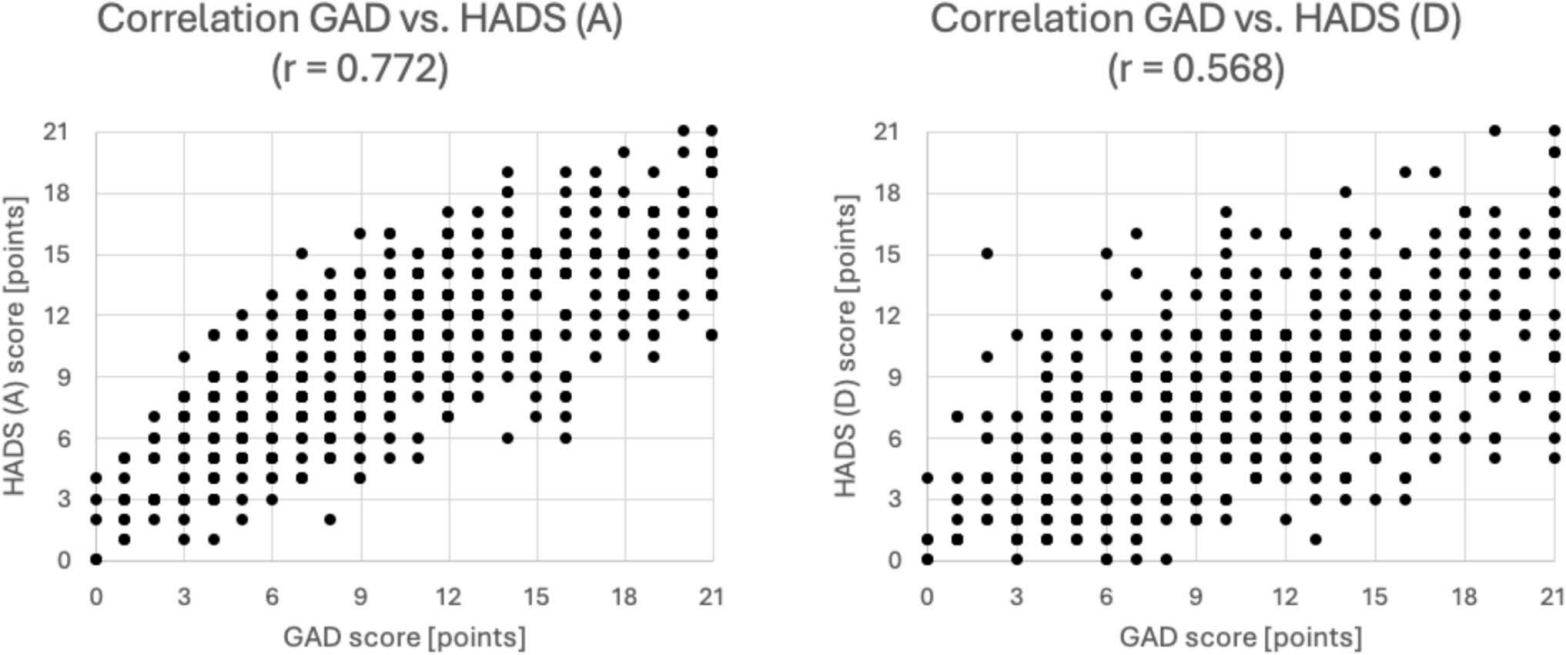
Correlation between the Generalized Anxiety Disorder questionnaire (GAD) and the Hospital Depression and Anxiety Score (HADS)

In 42.8% (251 participants) depression was not an associated factor with a normal HADS-Depression score (zero–seven points), while 29.5% (173 participants) had a chance of mild depression (eight–10 points) and 27.8% (163 participants) had a chance of moderate and severe depression (11–21 points). Only 24.0% (141 participants) had a normal HADS-Anxiety score (zero – seven points), indicating no association with anxiety disorders. 24.0% (141 participants) had a chance for mild anxiety (eight–10 points), while 52.0% percent (305 participants) had a chance for moderate to severe anxiety (11–21 points). The median score was 11.0 with an IQR of 6.0 for the HADS-A, and 8.0 with an interquartile range (IQR) of 6.0 for the HADS-D (Fig. 3).

The scatterplots show a significant correlation between HADS anxiety and depression scores (range zero to 21) and the GAD score (range zero to 21) (HADS-A r = 0.772, p < 0.001 (left), HADS-D r = 0.568, p < 0.001 (right).

### Sleep disorders were a common feature in PCOS

Less than 10% (9.8%, N = 56) of participants achieved a normal sleep score on the PSQI. 60.5% (N = 346) appeared to have mild sleep disturbance and 29.7% (N = 170) had chronic sleep disturbance. The median PSQI score was 9.0 with an IQR of 4.0.

**Fig. 4:**
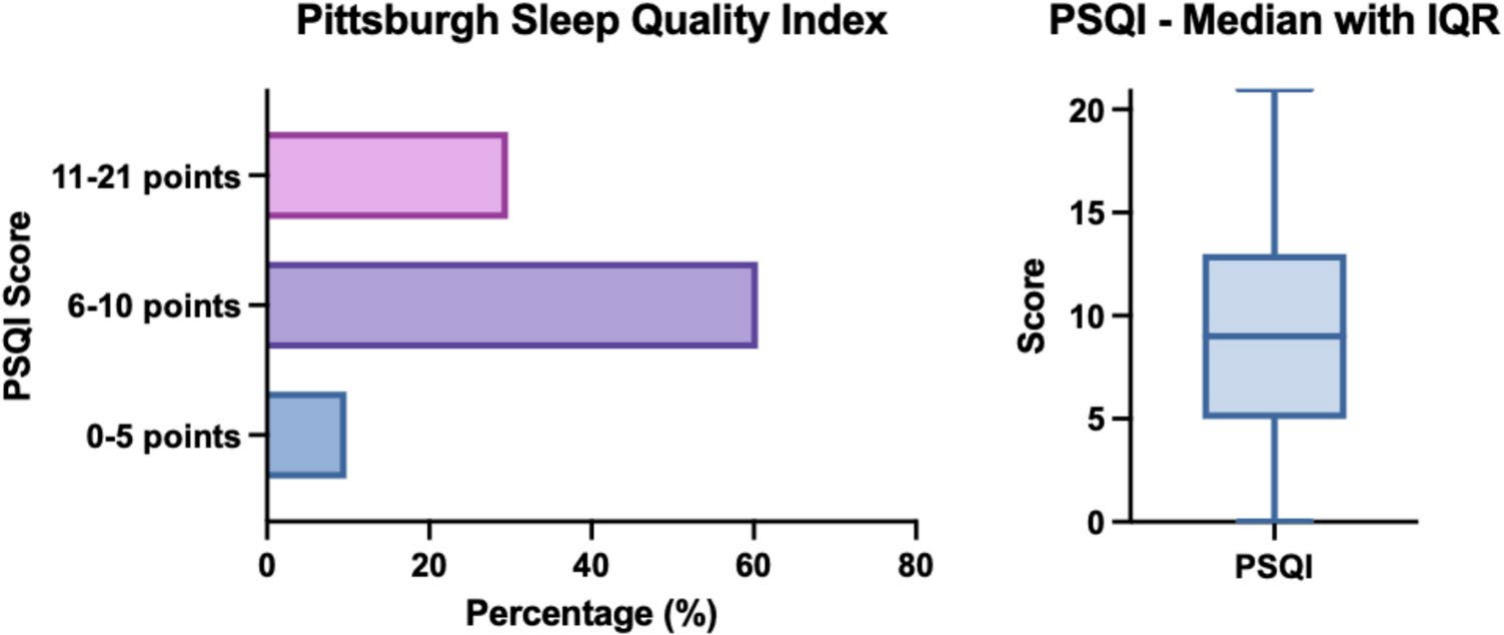
Frequency distribution of the Pittsburgh Sleep Quality Index (PSQI)

25.0% (N = 147) of participants rated their perceived sleep quality in the last four weeks as very poor and 8.7% (N = 51) had to take sleep medication (prescribed or over the counter) at least once a week. 13.1% (N = 77) took more than 60 min to fall asleep, 22.8% (N = 134) usually took 31–60 min to fall asleep each night. 28.2% (N = 164) slept more than seven hours per night in the past 30 days, 33.8% (N = 197) slept six to seven hours, 33.5% (N = 195) slept five to six hours and 4.5% (N = 26) slept less than five hours per night. Daytime dysfunction (defined as "difficulty staying awake while driving, eating, engaging in social activities" as well as "enthusiasm to get things done" [20]) was a severe problem for 14.1% (N = 83). Chronic sleep disturbance was an associated factor for anxiety and depression as well as OSA (p < 0.001) after adjustment for age (Fig. 4).

Only 9.8% (56 participants) achieved a normal score on the PSQI (< six points). 60.5% (N = 346) appeared to have mildly disturbed sleep (score six–10 points) and 29.7% (N = 170) suffered from chronic sleep disturbance (11–21 points). The median PSQI score was 9.0 with an interquartile range (IQR) of 4.0.

### OSA especially affected obese and insulin-resistant women with PCOS

**Fig. 5:**
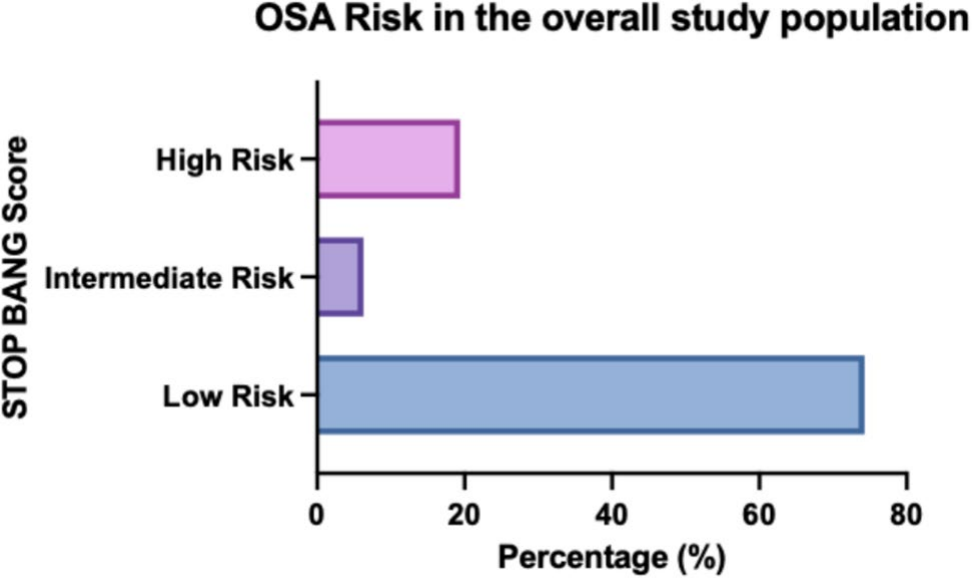
Probability of obstructive sleep apnoea (OSA) in the study population

**Fig. 6:**
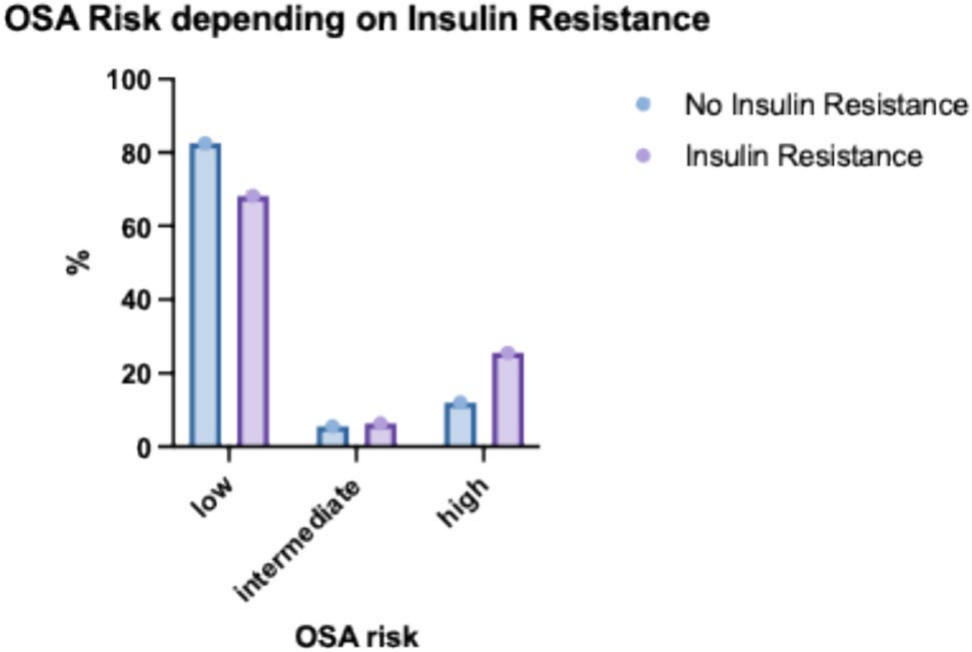
Probability of obstructive sleep apnea (OSA) according to insulin resistance

**Fig. 7:**
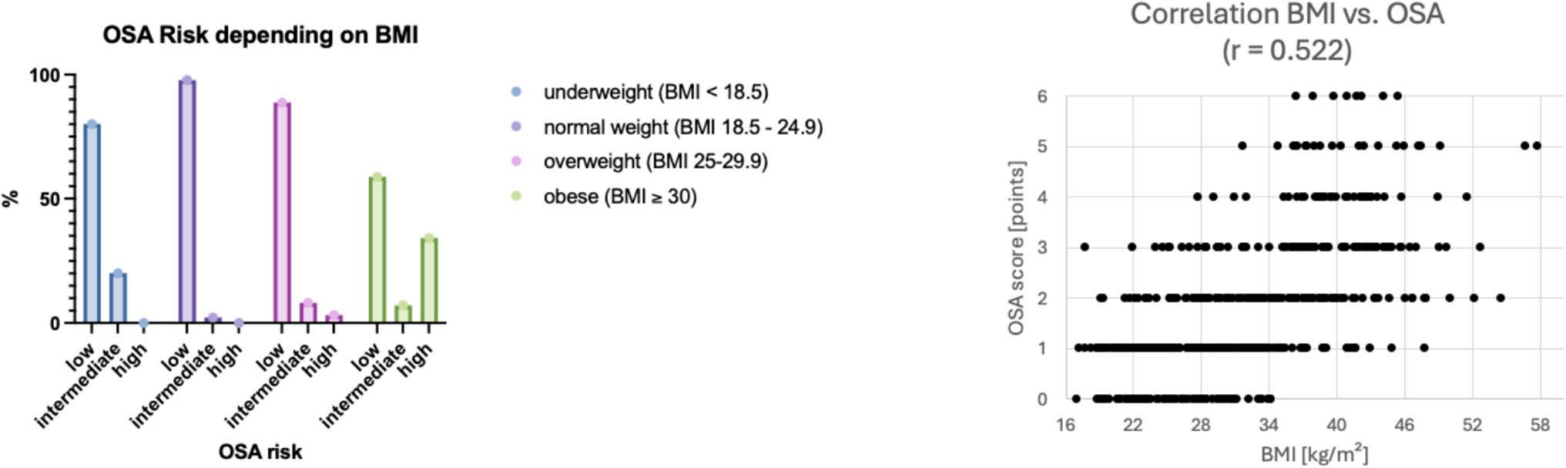
Probability of obstructive sleep apnea (OSA) according to body mass index (BMI)(left) and correlation between BMI and OSA (right)

19.5% of the study population (N = 114) had a high association with OSA according to the STOP BANG questionnaire. The association was significantly increased in overweight and obese women (BMI > 30 kg/m^2^) (37.3%, N = 114) (Fig. 5). There was a significant correlation between BMI (in kg/m^2^) and OSA association (r = 0.522, p < 0.001). Older age was also a significant predictor of OSA (p < 0.001). In addition, insulin resistance compared to normal glucose tolerance was identified as an associated factor for OSA in our study population. 25.5% (N = 68) of women with insulin resistance had a high chance for OSA compared to only 12.0% (N = 33) of women with normal glucose tolerance (p < 0.001) (Figs. 6, 7).

In the study population 74.3% (N = 436) had low probability of OSA, 6.3% (N = 37) were at intermediate risk and 19.4% (N = 114) had a high risk of developing or having OSA (Fig. 5).

Insulin resistance was an independent associated factor for OSA risk: 25.5% (N = 68) of women with insulin resistance were at high risk for OSA compared to only 12.0% (N = 33) of women with normal glucose tolerance (p < 0.001) (Fig. 6).

The probability of OSA was dependent on body mass index (p < 0.001). In particular, overweight and obese women had a high probability of OSA (37.3%, N = 114) compared to 0% (N = 0) in the underweight and normal weight group. The scatterplot (right) shows a significant correlation between BMI (in kg/m^2^) and OSA association (r = 0.522, p < 0.001) (Fig. 7).

## Discussion

This cross-sectional study supports our hypothesis that psychological comorbidities and sleep disturbances are common in women with PCOS. First, our study found a high association with anxiety and depression, which was BMI dependent. Second, the probability of sleep disorders and chronic sleep disturbance seemed to affect at least one third of women with PCOS. Thirdly, we showed that the chance of OSA was high in women with PCOS, particularly in overweight and obese individuals with insulin resistance.

A previous study reported a similar at-risk prevalence of anxiety and depression in an Australian cohort, which remained elevated after adjustment for BMI, socioeconomic factors and infertility [30]. Both conditions are common comorbidities in women affected with PCOS [5] and more than 50% may have at least one psychiatric disorder [12, 13]. Our study found a significant correlation between BMI, anxiety and depression. Especially in underweight and overweight/obese women anxiety or depression were common associated factors. This finding is supported by a previous meta-analysis that showed increased odds for depressive symptoms (OR 3.25, 95% CI 1.73–6.09) and anxiety symptoms (OR 6.30, 95% CI 1.88–21.09) in BMI-matched studies [14]. After adjustment for age and PSQI total score, which indicates chronic sleep disturbance, BMI did not significantly influence anxiety disorders in our study. We conclude that chronic sleep disturbance is also an associated factor for the development of anxiety symptoms in women with PCOS, independent of BMI, or vice versa: sleep disturbance may also be caused by anxiety [31, 32].

PCOS is associated with common sleep problems such as shorter sleep duration, poor sleep quality, fragmented sleep, difficulty falling asleep and feeling tired the next day [10, 11]. A meta-analysis of more than 16.000 participants found that about 16% of women with PCOS experienced sleep problems and they had a 6.22-fold risk of developing sleep disturbance compared to women without PCOS [10]. In comparison to these findings, we found a much higher prevalence of mild (60.5%) and chronic sleep disturbance (29.7%) in our study population. These data suggest that sleep disturbance is a more important comorbidity in women with PCOS than previously thought. We have also shown an association between sleep disturbance and anxiety, depression and OSA. Furthermore, poor sleep quality has been shown to have a negative impact on obesity, insulin resistance and cardiovascular health, creating a vicious cycle for women with PCOS [10, 33].

Women with PCOS have a higher prevalence of obstructive sleep apnea (OSA) than women without PCOS, regardless of BMI and age [34–36]. Primary risk factors for OSA in the general population are gender with a male to female ratio of 2:1, obesity, and age [9]. As hyperandrogenemia with elevated testosterone levels is a hallmark of PCOS and affected individuals are often obese, these two factors were thought to account for the high prevalence of OSA in PCOS women [37]. However, studies have shown that insulin resistance and glucose intolerance appear to be stronger indicators of the presence and severity of OSA in women with PCOS than BMI and androgen levels [35, 37]. Our findings support that insulin resistance is an independent associated factor for OSA besides age and BMI.

## Strengths and limitations

The data were collected through an online survey and were assumed to be truthful; however, the data were subjective. Biased participants may have exaggerated or minimized their symptoms depending on their experience. The diagnosis of PCOS was based on self-reported information, without confirmation through laboratory tests, ultrasound examinations or medical records. As the data collection was retrospective, memory bias may also have influenced the results. There was an under-representation of post-menopausal women in the study population, which could influence data on OSA association, as age is another important risk factor for the disease.

On the other hand, the online format, with distribution through social media and online platforms, enabled a large sample size to be generated, and women from different backgrounds and educational levels were recruited. Due to the high level of participation in the online study, the required number of participants was quickly exceeded, allowing for a much larger sample size than originally anticipated and increasing the statistical power of the study. The online survey format also eliminated investigator bias.

There was no control group, making it difficult to draw conclusions about the effect of PCOS on anxiety, depression and sleep problems compared to the general female population.

## Conclusion

Women with PCOS often struggle with anxiety and depression. Healthcare providers should be aware of the high prevalence and screen patients to refer them to specialists for treatment. Sleep disorders are very common and may exacerbate other comorbidities. Overweight and obese patients and women with insulin resistance may be particularly susceptible to OSA. Due to the multifaceted nature of PCOS, a holistic approach to treatment is essential for effective, patient-centered care.

## Supplementary Information

Below is the link to the electronic supplementary material.

Supplementary file1 (PDF 316 KB)

Supplementary file2 (DOCX 47 KB)

Supplementary file3 (XLSX 31 KB)

## Data Availability

Data is provided within the manuscript or supplementary information files. Remaining datasets generated during and/or analyzed during the current study are available from the corresponding author upon reasonable request.
