# Supplementary material for: Sleep disorders and psychological comorbidities in women with polycystic ovary syndrome – a cross-sectional study
Source: Arch Gynecol Obstet. 2025 May 13;312(2):573–82. doi: 10.1007/s00404-025-08049-9 (PMC12334535; doi:10.1007/s00404-025-08049-9)
Supplement: Supplementary file 1 — Supplementary file1 (PDF 316 KB) [file 404_2025_8049_MOESM1_ESM.pdf]

Kanton Bern
Canton de Berne

Gesundheits-, Sozial- und Integrationsdirektion
Kantonale Ethikkommission für die Forschung

Murtenstrasse 31
3010 Bern
Bern
+41 31 633 70 70 (Telefon)
+41 31 633 70 71 (Telefax)
info.kek@be.ch
Ethikkommission für die Forschung am Menschen (be.ch)

Dorothy Pfiffner
+41 31 633 70 77
dorothy.pfiffner@be.ch

GSI-KEK, Murtenstrasse 31, 3010 Bern

Frau
Prof. Dr. med. Petra Stute
Universitätsklinik für Frauenheilkunde
Theodor-Kocher-Haus
Friedbühlstrasse 19
3010 Bern

Bern, 30. Oktober 2023, CA

Clarification of jurisdiction

BASEC-Nr: Req-2023-01259 **Date of receipt:** 20/10/2023

Title: Lebensqualität, Körperbildwahrnehmung, psychische Komorbiditäten und Schlafstörungen bei Frauen mit Polyzstischem Ovarialsyndrom (PCOS)

Result of clarification of jurisdiction

☒ **Not responsible**: The project is not subject to ethical committee approval in Switzerland. Reason: The project does not fall under the Human Research Act, Art. 2, Paragraph 1.

☐ **Responsible:** Approval according to Human Research Act, Art. 2, Paragraph 1 is necessary in Switzerland. Please submit an application to the KEK according to www.swissethics.ch.

Fee: CHF 200.– (Tariff code x.x)

Date/Place: 30.10.2023/Bern

Prof. em. Dr. med. Christian Seiler
President

Dr. sc. nat. Dorothy Pfiffner
Head of the scientific secretariat
