# Supplementary material for: Sleep disorders and psychological comorbidities in women with polycystic ovary syndrome – a cross-sectional study
Source: Arch Gynecol Obstet. 2025 May 13;312(2):573–82. doi: 10.1007/s00404-025-08049-9 (PMC12334535; doi:10.1007/s00404-025-08049-9)
Supplement: Supplementary file 2 — Supplementary file2 (DOCX 47 KB) [file 404_2025_8049_MOESM2_ESM.docx]

Questionnaire

**1. I confirm, that I am at least 18 years old and have read and understood the consent form.**

O Yes

O No

2**. Have you previously got the diagnosis of prolactinoma or androgenital syndrome (AGS)? If one of these diagnoses has been made, participation in this study is not possible.**

O Yes, I’ve got of the diagnoses mentioned above.

O No, I don’t have any of the diagnoses mentioned above.

**3. What gender do you identify as?**

O female

O male

O diverse

O Not specified

**4. What is your year of birth?**

Free text answer

**5. Please specify your height in centimeters and your weight in kilograms.**

Free text answer

**6. What is your highest level of education?**

O student

O finished school without a diploma

O secondary school certificate

O diploma polytechnical school

O high school diploma/ university entrance qualification

O university degree

O other, please specify:

**7. Are you employed at the moment?**

O Yes, I am employed

O No, I am unemployed.

O No, I am retired.

O No, I am nothing from the above.

**8. What do you do professionally?**

O student

O doing an apprenticeship

O university student

O employee

O civil servant

O self-employed

O looking for work/ unemployed

O retirement

O other, please specify:

**9. My relationship status is currently…**

O single

O in a partnership

O married

O divorced

O widowed

O other/ no specification

**10. Do you live in a firm partnership?**

O no

O yes, since < 1 year

O yes, since > 1 year

O yes, since > 5 years

O yes, since > 10 years

**11. What is your country of residence?**

O Germany

O Austria

O Switzerland

O Other country, please specify:

**12. What is your country of birth?**

O Please specify:

**13. What is your parent’s country of birth?**

O Mother, please specify:

O Father, please specify:

**14. What ethnic origin do you identify with?**

O White

O person of color

O Black

O Jewish

O Russian jew

O Muslim

O Sinti and Roma

O Afrogerman

O Arabic

O Asian-German

O Polish-German

O Russian German

O Turkish- German

O I identify with: (Please specify)

**15. At what age did you first menstruate?**

O Age at first menstruation: (Please specify)

O I didn’t menstruate yet.

O I don’t know.

**16. Are you postmenopausal? (Postemenopause is specified as no menstruation > 12 months in women over 40 years.)**

O Yes, my last menstruation was ___ years ago.

O No.

**17. Are you pregnant or breast-feeding at the moment?**

O Yes

O No

**18. Was the start of your breast development more than 3 years ago?**

O Yes

O No

**19. Are you taking a hormonal contraceptive at the moment?**

O Yes

O No

**20. Which hormonal contraceptive are you taking at the moment?**

O combined oral contraceptive (“birth control pill”, COC)

O progestogen only pill (POP, “mini pill”)

O vaginal ring

O hormonal IUD (intrauterine device)

O contraceptive injection

O contraceptive implant

O contraceptive patch

O other, please specify:

**21. How long is your regular menstrual cycle?**

O Answer in days:

**22. How long is your regular menstruation (e.g. days, that you’re bleeding)?**

O Answer in days:

**23. How many menstrual cycles do you approximately have per year?**

O Answer in cycles per year:

**24. Did you ever have a menstrual cycle that lasted for more than 90 days?**

O Yes

O No

**25. Do you have or did you ever have problems with acne?**

O Yes

O No

**26. Do you have or did you ever have problems with excessive hair loss?**

O Yes

O No

**27. To further specify excessive hair loss, you’ll see a picture of different types of severity. Which type are you?** INSERT Images

O Type 1

O Type 2

O Type 3

**28. Do you have or did you ever have problems with excessive body hair?**

O Yes

O No

O I don’t know.

**29. In the following picures you will see different severity types of excessive body hair for different body areas. Please specify, which type fits the most for your hair in this body area. (Assuming that you haven’t removed body hair through shaving or other procedures.)**

INSERT Images

**33. Have you ever had the male sexual hormones in your blood measured?**

O Yes

O No

**34. Has an increased level of male hormones been detected in the process?**

O Yes

O No

**35. Have you ever had an ultrasound of the ovaries?**

O Yes

O No

**36. Have enlarged ovaries been detected?**

O Yes

O No

**37. Have an increased number of follicles (sometimes also called cysts) been detected?**

O Yes

O No

**38. Has a doctor ever told you that you have polycystic ovary syndrome (PCOS)?**

O Yes

O No

**39. Have you ever had a blood glucose test?**

O Yes

O No

**40. Do you have any of the following conditions?**

O Diabetes mellitus

O Gestational diabetes (current or in the past)

O Pre-diabetes (e.g., insulin resistance)

O None of the above

**41. Which of the following options have you tried with regard to your elevated sugar levels?**

Several answers are possible

O Lifestyle changes (diet, exercise, etc.)

O Medication

Please check the appropriate medication:

Several answers are possible

O Metformin

O other diabetes tablets

O Insulin (syringes/pen)

O other

Bariatric operations (e.g., gastric bypass)

Others, please elaborate: ______

**42. Which of the measures are you currently still carrying out?**

Several answers can be selected.

O Lifestyle changes (diet, exercise, etc.)

O Medication

**Please check the appropriate medication:**

O Metformin

O other diabetes tablets

O Insulin (syringes/pen)

O other

O psychotherapy

O Bariatric operations (e.g., gastric bypass)

O Others. __________

None

**43. Do you have any other known pre-existing conditions?**

Also consider illnesses that are not directly related to gynecology. For example, high blood pressure, diabetes, elevated blood lipid levels.

O Yes

**What pre-existing conditions do you have?** Please specify: ________

O No

**44. Are you currently taking medication?**

O Yes

O No

**45. What medication are you taking? Please specify:**

If possible, write down the exact preparation and its dosage, e.g.: Metoprolol 95mg once in the morning: _________________________

**46. Do you smoke? Cigarettes, e-cigarettes, water pipes, etc**

O Yes

O No

**47. Do you smoke more than 15 cigarettes a day? (Or equivalent to 15 cigarettes)**

O Yes

O No

**48. Would you like to have children one day?**

O Yes

O No

**49. Approximately how long have you been planning to have children?**

O 1 month

O 1-3 months

O 3-6 months

O 6-9 months

O 9-12 months

O 1-2 years

O 2-3 years

O 3-4 years

O more than 4 years

O unknown

**50. Have you ever tried to get pregnant for over a year without success?**

Trying to get pregnant = regular, unprotected sexual intercourse at the right time

O Yes

O No

**51. Are you currently undergoing fertility treatment?**

O Yes

O No

**52. How long have you been undergoing fertility treatment?** __________

**53. To what extent have you felt that growth of visible hair on your chin has been a problem for you during the last two weeks:**

A Severe A Major A Moderate Some A Little Hardly any No

Problem Problem Problem Problem Problem Problem Problem

Growth of visible hair on chin? O O O O O O O

**54. During the past two weeks, how much of the time have you felt:**

All of the Most of the A Good Bit Some of the A Little of Hardly any None of

Time Time of the Time Time the Time of the Time the Time

Depressed as O O O O O O O

a result of

having PCOS?

Concerned O O O O O O O

about being

overweight?

Easily tired? O O O O O O O

Concerned O O O O O O O

with infertility

problems?

Moody as a O O O O O O O

result of having

PCOS

**55. In relation to you last menstruation, how much were the following issues a problem for you:**

A Severe A Major A Moderate Some A Little Hardly any No

Problem Problem Problem Problem Problem Problem Problem

Headaches? O O O O O O O

Irregular O O O O O O O

menstrual

periods?

**56. To what extent has growth of visible hair on your upper lip been a problem for you during the last two weeks:**

A Severe A Major A Moderate Some A Little Hardly any No

Problem Problem Problem Problem Problem Problem Problem

Growth of O O O O O O O

visible hair

on upper

lip?

**57. During the past two weeks, how much of the time have you:**

All of the Most of the A Good Bit Some of the A Little of Hardly any None of

Time Time of the Time Time the Time of the Time the Time

Had trouble O O O O O O O

dealing with your

weight?

Had low self-esteem O O O O O O O

As a result

of having your

PCOS?

Felt frustration O O O O O O O

in trying to lose

weight?

Felt afraid of O O O O O O O

not being able to

have children?

Felt frightened O O O O O O O

of getting cancer?

**58. Over the last two weeks, to what extent the following issues have been a problem for you:**

A Severe A Major A Moderate Some A Little Hardly any No

Problem Problem Problem Problem Problem Problem Problem

Growth of visible O O O O O O O

hair on your face?

Embarrassment O O O O O O O

about excessive

body hair?

**59. During the past two weeks how much of the time have you been:**

All of the Most of the A Good Bit Some of the A Little of Hardly any None of

Time Time of theTime Time the Time of the Time the Time

Worried O O O O O O O

about having

PCOS?

Self-conscious O O O O O O O

as a result of having

PCOS?

**60. In relation to your last menstruation, how much the following issues were a problem for you:**

A Severe A Major A Moderate Some A Little Hardly any No

Problem Problem Problem Problem Problem Problem Problem

Abdominal O O O O O O O

Bloating?

Late menstrual O O O O O O O

period?

Menstrual O O O O O O O

cramps?

**61. How much of the time during the last two weeks did you:**

All of the Most of the A Good Bit Some of the A Little of Hardly any None of

Time Time of the Time Time the Time of the Time the Time

Feel like you O O O O O O O

are not sexy

because of being

overweight?

Feel a lack of O O O O O O O

control over the

situation with

PCOS?

Have O O O O O O O

difficulties staying

at your ideal

weight?

Feel sad O O O O O O O

because of

infertility

problems?

**62. To what extent has growth of visible body hair been a problem for you during**

**the last two weeks:**

A Severe A Major A Moderate Some A Little Hardly any No

Problem Problem Problem Problem Problem Problem Problem

Growth of visible O O O O O O O

body hair?

**63. To what extent was acne a problem for you in the last two weeks?**

A Severe A Major A Moderate Some A Little Hardly any No

Problem Problem Problem Problem Problem Problem Problem

Acne? O O O O O O O

**64. In relation to your last menstruation, how much was acne a problem for you?**

A Severe A Major A Moderate Some A Little Hardly any No

Problem Problem Problem Problem Problem Problem Problem

Acne? O O O O O O O

**65. How much time during the last two weeks …**

All of the Most of the A Good Bit Some of the A Little of Hardly any None of

Time Time of the Time Time the Time of the Time the Time

.. did you feel O O O O O O O

unattractive

because of acne?

… did you feel O O O O O O O

depressed

as a result of acne?

*Choose the reply that is closest to how you have been feeling* ***in the past week****. Don't take too long over you replies: your immediate is best.*

**66. I feel tense or 'wound up':**

O Most of the time

O A lot of the time

O From time to time

O Not at all

**67. I still enjoy the things I used to enjoy:**

O Definitely as much

O Not quite so much

O Only a little

O Hardly at all

**68. I get a sort of frightened feeling as if something awful is about to happen:**

O Very definitely and quite badly

O Yes, but not too badly

O A little, but it doesn't worry me

O Not at all

**69. I can laugh and see the funny side of things:**

O As much as I always could

O Not quite so much now

O Definitely not so much now

O Not at all

**70. Worrying thoughts go through my mind:**

O A great deal of the time

O A lot of the time

O From time to time, but not too often

O Only occasionally

**71. I feel cheerful:**

O Not at all

O Not often

O Sometimes

O Most of the time

**72. I can sit at ease and feel relaxed:**

O Definitely

O Usually

O Not Often

O Not at all

**73. I feel as if I am slowed down:**

O Nearly all the time

O Very often

O Sometimes

O Not at all

**74. I get a sort of frightened feeling like 'butterflies' in the stomach:**

O Not at all

O Occasionally

O Quite Often

O Very Often

**75. I have lost interest in my appearance:**

O Definitely

O I don't take as much care as I should

O I may not take quite as much care

O I take just as much care as ever

**76. I feel restless as I have to be on the move:**

O Very much indeed

O Quite a lot

O Not very much

O Not at all

**77. I look forward with enjoyment to things:**

O As much as I ever did

O Rather less than I used to

O Definitely less than I used to

O Hardly at all

**78. I get sudden feelings of panic:**

O Very often indeed

O Quite often

O Not very often

O Not at all

**79. I can enjoy a good book or radio or TV program:**

O Often

O Sometimes

O Not often

O Very seldom

*Please indicate below how much the statements apply to you.*

**80. I feel that I am a person of worth, at least on an equal plane with others.**

O Strongly agree

O Agree

O Disagree

O Strongly disagree

**81. I feel that I have a number of good qualities.**

O Strongly agree

O Agree

O Disagree

O Strongly disagree

**82. All in all, I am inclined to feel that I am a failure.**

O Strongly agree

O Agree

O Disagree

O Strongly disagree

**83. I am able to do things as well as most other people.**

O Strongly agree

O Agree

O Disagree

O Strongly disagree

**84. I feel I do not have much to be proud of.**

O Strongly agree

O Agree

O Disagree

O Strongly disagree

**85. I take a positive attitude toward myself.**

O Strongly agree

O Agree

O Disagree

O Strongly disagree

**86. On the whole, I am satisfied with myself.**

O Strongly agree

O Agree

O Disagree

O Strongly disagree

**87. I wish I could have more respect for myself.**

O Strongly agree

O Agree

O Disagree

O Strongly disagree

**88. I certainly feel useless at times.**

O Strongly agree

O Agree

O Disagree

O Strongly disagree

**89. At times I think I am no good at all.**

O Strongly agree

O Agree

O Disagree

O Strongly disagree

*The following pages contain a series of statements about how people might think, feel, or behave. You are asked to indicate the extent to which each statement pertains to you personally. In order to complete the questionnaire, read each statement carefully and decide how much it pertains to you personally. There are no right or wrong answers. Just give the answer that is most accurate for you. Remember, your responses are confidential, so please be completely honest and answer all items.*

**90. Before going out in public, I always notice how I look.**

O Definitely disagree

O Mostly disagree

O Neither agree nor disagree

O Mostly agree

O Definitely agree

**91. I am careful to buy clothes that will make me look my best.**

O Definitely disagree

O Mostly disagree

O Neither agree nor disagree

O Mostly agree

O Definitely agree

**92. My body is sexually appealing.**

O Definitely disagree

O Mostly disagree

O Neither agree nor disagree

O Mostly agree

O Definitely agree

**93. I constantly worry about being or becoming fat.**

O Definitely disagree

O Mostly disagree

O Neither agree nor disagree

O Mostly agree

O Definitely agree

**94. I like my looks just the way they are.**

O Definitely disagree

O Mostly disagree

O Neither agree nor disagree

O Mostly agree

O Definitely agree

**95. I check my appearance in a mirror whenever I can.**

O Definitely disagree

O Mostly disagree

O Neither agree nor disagree

O Mostly agree

O Definitely agree

**96. Before going out, I usually spend a lot of time getting ready.**

O Definitely disagree

O Mostly disagree

O Neither agree nor disagree

O Mostly agree

O Definitely agree

**97. I am very conscious of even small changes in my weight.**

O Definitely disagree

O Mostly disagree

O Neither agree nor disagree

O Mostly agree

O Definitely agree

**98. Most people would consider me good-looking.**

O Definitely disagree

O Mostly disagree

O Neither agree nor disagree

O Mostly agree

O Definitely agree

**99. It is important that I always look good.**

O Definitely disagree

O Mostly disagree

O Neither agree nor disagree

O Mostly agree

O Definitely agree

**100. I use very few grooming products.**

O Definitely disagree

O Mostly disagree

O Neither agree nor disagree

O Mostly agree

O Definitely agree

**101. I like the way I look without my clothes on.**

O Definitely disagree

O Mostly disagree

O Neither agree nor disagree

O Mostly agree

O Definitely agree

**102. I am self-conscious if my grooming isn't right.**

O Definitely disagree

O Mostly disagree

O Neither agree nor disagree

O Mostly agree

O Definitely agree

**103. I usually wear whatever is handy without caring how it looks.**

O Definitely disagree

O Mostly disagree

O Neither agree nor disagree

O Mostly agree

O Definitely agree

**104. I like the way my clothes fit me.**

O Definitely disagree

O Mostly disagree

O Neither agree nor disagree

O Mostly agree

O Definitely agree

**105. I don't care what people think about my appearance.**

O Definitely disagree

O Mostly disagree

O Neither agree nor disagree

O Mostly agree

O Definitely agree

**106. I take special care with my hair grooming.**

O Definitely disagree

O Mostly disagree

O Neither agree nor disagree

O Mostly agree

O Definitely agree

**107. I dislike my physique.**

O Definitely disagree

O Mostly disagree

O Neither agree nor disagree

O Mostly agree

O Definitely agree

**108. I am physically unattractive.**

O Definitely disagree

O Mostly disagree

O Neither agree nor disagree

O Mostly agree

O Definitely agree

**109. I never think about my appearance.**

O Definitely disagree

O Mostly disagree

O Neither agree nor disagree

O Mostly agree

O Definitely agree

**110. I am always trying to improve my physical appearance.**

O Definitely disagree

O Mostly disagree

O Neither agree nor disagree

O Mostly agree

O Definitely agree

**111. I am on a weight-loss diet.**

O Definitely disagree

O Mostly disagree

O Neither agree nor disagree

O Mostly agree

O Definitely agree

**112. I have tried to lose weight by fasting or going on crash diets.**

O Never

O Rarely

O Sometimes

O Often

O Very Often

**113. I think I am:**

O Very Underweight

O Somewhat Underweight

O Normal Weight

O Somewhat Overweight

O Very Overweight

**114. From looking at me, most other people would think I am:**

1. Very Underweight

2. Somewhat Underweight

3. Normal Weight

4. Somewhat Overweight

5. Very Overweight

*Use this scale to indicate how dissatisfied or satisfied you are with each of the following areas or aspects of your body:*

**115. Face (facial features, complexion)**

O Very satisfied

O Mostly satisfied

O Neither satisfied nor dissatisfied

O Mostly satisfied

O Very satisfied

**116. Hair (color, thickness, texture)**

O Very satisfied

O Mostly satisfied

O Neither satisfied nor dissatisfied

O Mostly satisfied

O Very satisfied

**117. Lower torso (buttocks, hips, thighs, legs)**

O Very satisfied

O Mostly satisfied

O Neither satisfied nor dissatisfied

O Mostly satisfied

O Very satisfied

**118. Mid torso (waist, stomach)**

O Very satisfied

O Mostly satisfied

O Neither satisfied nor dissatisfied

O Mostly satisfied

O Very satisfied

**119. Upper torso (chest or breasts, shoulders, arms)**

O Very satisfied

O Mostly satisfied

O Neither satisfied nor dissatisfied

O Mostly satisfied

O Very satisfied

**120. Muscle tone**

O Very satisfied

O Mostly satisfied

O Neither satisfied nor dissatisfied

O Mostly satisfied

O Very satisfied

**121. Weight**

O Very satisfied

O Mostly satisfied

O Neither satisfied nor dissatisfied

O Mostly satisfied

O Very satisfied

**122. Height**

O Very satisfied

O Mostly satisfied

O Neither satisfied nor dissatisfied

O Mostly satisfied

O Very satisfied

**123. Overall appearance**

O Very satisfied

O Mostly satisfied

O Neither satisfied nor dissatisfied

O Mostly satisfied

O Very satisfied

**124. Please answer the following questions below to determine if you might be at risk.**
**a. Do you Snore Loudly (loud enough to be heard through closed doors or your bed-partner elbows you for snoring at night)?**

O Yes

O No

**b. Do you often feel Tired, Fatigued, or Sleepy during the daytime (such as falling asleep during driving or talking to someone)?**

O Yes

O No

**c. Has anyone Observed you Stop Breathing or Choking/Gasping during your sleep ?**

O Yes

O No

**d. Do you have or are being treated for High Blood Pressure ?**

O Yes

O No

**e. Body Mass Index more than 35 kg/m2?**

Height:

Weight:

BMI:

O Yes

O No

**f. Age older than 50 ?**

O Yes

O No

**g. Neck size large ? (Measured around Adams apple) Is your shirt collar 16 inches / 41cm or larger?**

O Yes

O No

**h. Gender = Male ?**

O Yes

O No

**125. Over the last two weeks, how often have you been bothered by any of the following problems?**

**a. Feeling nervous, anxious or on edge?**

O Not at all

O Several days

O More than half the days

O Nearly every day

**b. Not being able to stop or control worrying?**

O Not at all

O Several days

O More than half the days

O Nearly every day

**c. Worrying too much about different things?**

O Not at all

O Several days

O More than half the days

O Nearly every day

**d. Trouble relaxing?**

O Not at all

O Several days

O More than half the days

O Nearly every day

**e. Being so restless that it is hard to sit still?**

O Not at all

O Several days

O More than half the days

O Nearly every day

**f. Becoming easily annoyed or irritable?**

O Not at all

O Several days

O More than half the days

O Nearly every day

**g. Feeling afraid as if something awful might happen?**

O Not at all

O Several days

O More than half the days

O Nearly every day

**126. The following questions relate to your usual sleep habits during the past month only. Your answers should indicate the most accurate reply for the majority of days and nights in the past month. Please answer all questions.**

During the past month, what time have you usually gone to bed at night? ___________________

During the past month, how long (in minutes) has it usually taken you to fall asleep each night? __________

During the past month, what time have you usually gotten up in the morning? ___________________

During the past month, how many hours of actual sleep did you get at night? (This may be different than the number of hours you spent in bed.) ___________________

**During the past month, how often have you had trouble sleeping because you...**

a. Cannot get to sleep within 30 minutes

O Not during the past month
O Less than once a week
O Once or twice a week
O Three or more times a week

b. Wake up in the middle of the night or early morning

O Not during the past month
O Less than once a week
O Once or twice a week
O Three or more times a week

c. Have to get up to use the bathroom

O Not during the past month
O Less than once a week
O Once or twice a week
O Three or more times a week

d. Cannot breathe comfortably

O Not during the past month
O Less than once a week
O Once or twice a week
O Three or more times a week

e. Cough or snore loudly

O Not during the past month
O Less than once a week
O Once or twice a week
O Three or more times a week

f. Feel too cold g

O Not during the past month
O Less than once a week
O Once or twice a week
O Three or more times a week

g. Feel too hot

O Not during the past month
O Less than once a week
O Once or twice a week
O Three or more times a week

h. Have bad dreams

O Not during the past month
O Less than once a week
O Once or twice a week
O Three or more times a week

i. Have pain

O Not during the past month
O Less than once a week
O Once or twice a week
O Three or more times a week

j. Other reason(s), please describe:

O Not during the past month
O Less than once a week
O Once or twice a week
O Three or more times a week

**During the past month, how often have you taken medicine to help you sleep (prescribed or “over the counter”)?**

O Not during the past month
O Less than once a week
O Once or twice a week
O Three or more times a week

**During the past month, how often have you had trouble staying awake while driving, eating meals, or engaging in social activity?**

O Not during the past month
O Less than once a week
O Once or twice a week
O Three or more times a week

**During the past month, how much of a problem has it been for you to keep up enough enthusiasm to get things done?**

O No problem at all
O Only a very slight problem
O Somewhat of a problem
O A very big problem

**During the past month, how would you rate your sleep quality overall?**

O Very good
O Fairly good
O Fairly bad
O Very bad

**Do you have a bed partner or room mate?**

O No bed partner or room mate
O Partner/room mate in other room
O Partner in same room but not same bed
O Partner in same bed

**If you have a room mate or bed partner, ask him/her how often in the past month you have had:**

a. Loud snoring

O Not during the past month
O Less than once a week
O Once or twice a week
O Three or more times a week

b. Long pauses between breaths while asleep

O Not during the past month
O Less than once a week
O Once or twice a week
O Three or more times a week

c. Legs twitching or jerking while you sleep

O Not during the past month
O Less than once a week
O Once or twice a week
O Three or more times a week

d. Episodes of disorientation or confusion during sleep

O Not during the past month
O Less than once a week
O Once or twice a week
O Three or more times a week

e. Other restlessness while you sleep, please describe:

O Not during the past month
O Less than once a week
O Once or twice a week
O Three or more times a week
